# The future of biomolecular simulation in the pharmaceutical industry: what we can learn from aerodynamics modelling and weather prediction. Part 1. understanding the physical and computational complexity of *in silico* drug design

**DOI:** 10.1107/S2059798321009712

**Published:** 2021-10-27

**Authors:** Tom Edwards, Nicolas Foloppe, Sarah Anne Harris, Geoff Wells

**Affiliations:** a School of Molecular and Cellular Biology, University of Leeds, Leeds, United Kingdom; b Astbury Centre for Structural and Molecular Biology, University of Leeds, Leeds, United Kingdom; c Vernalis (R&D) Ltd, Cambridge, United Kingdom; d School of Physics and Astronomy, University of Leeds, Leeds, United Kingdom; e School of Pharmacy, University College London, London, United Kingdom

**Keywords:** biomolecular simulation, molecular docking, *in silico* drug design, pharmaceutical industry

## Abstract

The predictive power of simulation has become embedded in the infrastructure of modern economies. Computer-aided design is ubiquitous throughout industry. In aeronautical engineering, built infrastructure and materials manufacturing, simulations are routinely used to compute the performance of potential designs before construction. The ability to predict the behaviour of products is a driver of innovation by reducing the cost barrier to new designs, but also because radically novel ideas can be piloted with relatively little risk. Accurate weather forecasting is essential to guide domestic and military flight paths, and therefore the underpinning simulations are critical enough to have implications for national security. However, in the pharmaceutical and biotechnological industries, the application of computer simulations remains limited by the capabilities of the technology with respect to the complexity of molecular biology and human physiology. Over the last 30 years, molecular-modelling tools have gradually gained a degree of acceptance in the pharmaceutical industry. Drug discovery has begun to benefit from physics-based simulations. While such simulations have great potential for improved molecular design, much scepticism remains about their value. The motivations for such reservations in industry and areas where simulations show promise for efficiency gains in preclinical research are discussed. In this, the first of two complementary papers, the scientific and technical progress that needs to be made to improve the predictive power of biomolecular simulations, and how this might be achieved, is firstly discussed (Part 1). In Part 2, the status of computer simulations in pharma is contrasted with aerodynamics modelling and weather forecasting, and comments are made on the cultural changes needed for equivalent computational technologies to become integrated into life-science industries.

## Abstract 2

The predictive power of simulation has become embedded in the infrastructure of modern economies. The scientific and technical progress that needs to be made to improve the predictive power of biomolecular simulations and how this might be achieved are discussed.

## 1. The physical properties of biomolecules and how this relates to their function   

In biochemistry, the accepted paradigm is that structure underpins function. Structural biology provides the basis for our understanding of biological mechanisms, including diseases caused by mutations and infection, and the design of potential therapies. The concept of a specific binding pocket with the correct shape and chemical complementarity to accommodate a drug (embraced by the ‘lock-and-key hypothesis’ of biomolecular interactions; Lemieux & Spohr, 1994 ▸) is central to rational structure-based design. The Protein Data Bank contains over 180 000 biomolecular structures (as of May 2021) determined by X-ray, NMR and electron cryo-microscopy (cryo-EM) to atomic resolution (https://www.rcsb.org; Burley *et al.*, 2021 ▸). While this is invaluable for investigating biomolecular recognition within complexes, it is often not sufficient to translate the biochemical ‘structure–function’ concept into a quantitative algorithm for predicting how strongly a putative drug will bind to its target protein and whether it would disrupt functional protein–protein inter­actions. Nevertheless, the observation that pharmaceutical companies run automated downloads of the PDB, as well as often performing in-house structure determination using X-ray crystallography and now increasingly cryo-EM, shows the value of this structural information to structure-based drug design.

**Figure 1:**
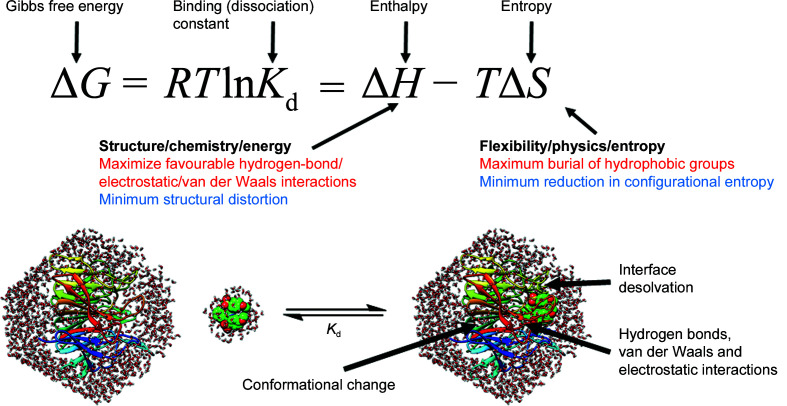
The changes in free energy (Δ*G*) that drive molecular recognition. The equilibrium is biased towards ligand binding when the thermodynamically favourable interactions (for example electrostatic attraction, hydrogen bonding, burial of hydrophobic groups and van der Waals forces) are larger than the thermodynamically unfavourable contributions (for example ligand desolvation, reduction in entropy associated with complexation and structural distortion of the ligand or protein, for example during induced-fit interactions).

The relationship between the static structures of interacting partners and their binding affinities is, however, obscured by the dynamic nature of biomolecules. Proteins are sufficiently deformable that they are classified as ‘soft matter’. As a consequence of this soft mechanics, thermal motion generates molecular conformations that can differ substantially from the average structure. This ensemble of conformations is very difficult to observe experimentally, as are the rearrangements of water networks that take place during molecular recognition. Measurements of biomolecular affinities performed using techniques such as isothermal titration calorimetry (ITC; Huddler & Zartler, 2017 ▸) or microscale thermophoresis (Jerabek-Willemsen *et al.*, 2014 ▸) probe the average interaction between ∼10^13^ biomolecules over timescales of seconds to minutes under environmental conditions that are frequently different from those required for experimental structural studies. Solvent interactions, especially hydrophobic effects, are central to biomolecular recognition, but remain poorly understood at the structural level. The balance between structure and dynamics in biomolecular recognition is captured by the thermodynamic definition of the free energy (Δ*G*; see Fig. 1 ▸), which is directly related to the binding affinity. Additional understanding of the underlying molecular changes can be obtained by performing ITC at different temperatures and measuring heat-capacity changes (Δ*C*_p_). While Δ*C*_p_ is broadly correlated with the degree of burial of apolar surfaces on complexation, other factors such as changes in protein flexibility during induced fit, or salt concentration, can make a significant contribution (Bergqvist *et al.*, 2004 ▸).

The soft mechanics of biomolecules is vital to their function. It enables them to act as molecular switches and machines. The mixture of stiff, ordered secondary structure and flexible disordered loops and hinges in proteins generates a complex underlying free-energy landscape containing multiple energy minima separated by energy barriers. In biomolecular switches, the binding of a specific activator or repressor perturbs the shape of this free-energy landscape to favour a new conformation. Allosteric communication, signalling cascades, cell membrane transporters and molecular motors all perform their functions by undergoing large conformational changes in response to the binding of other proteins or metabolites. It is often difficult to characterize all of the important states using structural studies of fixed species, particularly for membrane proteins (see Table 1 ▸).

The solvent/lipid membrane environment has an enormous effect on protein kinetic timescales, as well as on protein structure and thermodynamics. The ratio of the inertial to viscous forces (the Reynolds number) for proteins in water is extremely low, meaning that protein motions are heavily overdamped. Overcoming multiple free-energy barriers in a highly viscous environment requires time. Consequently, proteins explore their conformational states very slowly, with implications for relevant simulation timescales.

## 2. Physics-based biomolecular simulation for pharmaceutical design: successes and limitations   

**Figure 2:**
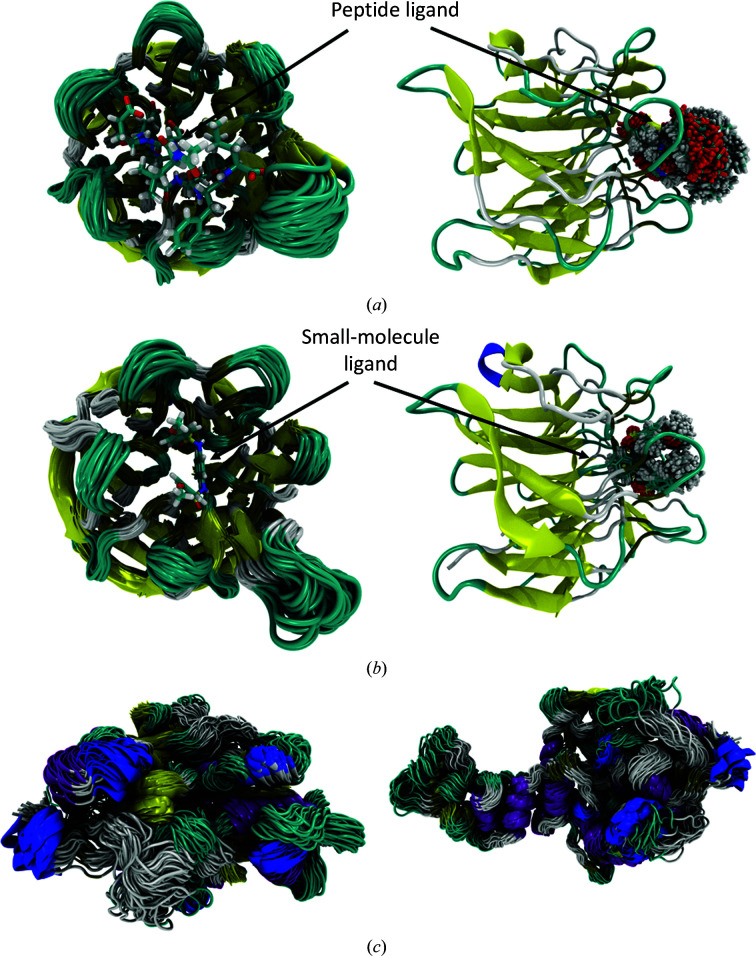
Protein dynamics for (*a*) a Keap1 Kelch domain–peptide complex (left panel; PDB entry 2flu) and (*b*) a Keap1–small-molecule complex (left panel; PDB entry 4iqk); ligand dynamics for the systems are shown in the right panels and a movie of the Keap1–small-molecule complex is provided as supporting information. (*c*) The SARS-CoV-2 nsp13 helicase protein modelled from the SARS-CoV-1 structure (PDB entry 4jyt) is shown in the left (top view) and right (side view) panels. Each of the images shows dynamics sampled every 10 ns from 1 µs trajectories. Protein structures are shown in a cartoon representation, coloured by secondary structure, and the ligands (left and centre) are shown in liquorice representation, coloured by atom name, and indicated with arrows. Images were created using *VMD*. Over femtosecond to picosecond timescales, the main motion is atomic bond vibrations and local side-chain rearrangements. Over longer (nanosecond to microsecond) timescales, the protein and ligand undergo large-scale, overdamped, global motions around a free-energy minimum. Proteins have complex free-energy landscapes containing multiple minima, which give rise to different conformational states which may be functionally relevant. Over extended (microsecond to millisecond) timescales, the protein will diffuse between these conformations. Over even longer timescales, the ligand will repeatedly bind and unbind from the pocket.

Biomolecular simulation provides dynamic information to better connect static experimental structures to biological function. These ‘physics-based’ simulations rely on two fundamental ingredients which both introduce approximations and caveats into the computer models. The first is the calculation of conformational energies via a set of empirical potentials known as the ‘force field’ and the second is sampling the numerous configurations of a molecular system, including solute conformations and solvent motions. Not all sampling methods are suitable to compute physically sound quantities; currently, molecular dynamics (MD) under defined temperature and pressure has emerged as the main strategy, although Monte Carlo-based probabilistic sampling has been used for coarse-grained modelling (Ouldridge *et al.*, 2011 ▸; Kmiecik *et al.*, 2016 ▸) and to improve the efficiency of relative binding free-energy calculations (Cournia *et al.*, 2017 ▸). MD simulations use Newtonian mechanics to evolve biomolecular conformations as a function of time, often in full atomic detail, thereby generating an ensemble of molecular structures that arises due to thermal fluctuations, as shown in Fig. 2 ▸. Water can be represented explicitly, so the fluctuating water networks that drive hydrophobic interactions are accounted for, and charged counterions can be included.

Molecular-dynamics simulations have become almost routine, and the availability of fast and cheap GPU processing has made them accessible to researchers who do not have access to high-performance computing (HPC) facilities. An overview of the field has been published by the Collaborative Computational Project for Biomolecular Simulation (CCPBioSim; Huggins *et al.*, 2019 ▸). A comprehensive collection of accessible reviews of current MD topics, such as advanced sampling, force-field development and the inclusion of experimental data (Bonomi & Camilloni, 2019 ▸), and expert articles describing state of the art in computational drug design (Wade & Salo-Ahen, 2019 ▸) are also available.

In principle, MD simulations have the potential to allow us to observe the binding equilibria between ligands and their biomolecular targets, to obtain on/off rates and binding affinities, and to predict large-scale protein conformational changes in response to external stimuli, such as effector or cofactor binding. In practice, however, the speed of the algorithms and the accuracy of the simulations are both still sufficiently limited that the calculations are not fully predictive, for the reasons discussed in detail below (Sections 2.1 and 2.2). The opportunities for improving the quantitative predictions of biomolecular simulation are summarized in Table 2 ▸.

### 2.1. Conformational sampling is limited by computational cost   

An atomistic MD simulation of a protein is typically performed over microsecond timescales, which may take around one month of simulation time depending on the size of the protein and the computational resources available. The constraints of the shortest length scale in an atomistic simulation (usually covalent bonds to hydrogen) place a strict upper limit on the integration time step that can be used to evolve the dynamic trajectory. The timescale of MD simulations is restricted by the dual constraints of this short time step and the maximal speed achievable per step. As a time step of 2 fs is common, a typical simulation requires 10^9^ MD cycles. Unfortunately, increasing the number of processors used to run the simulation can only improve the speed up to a hard limit. Eventually, the communication time required to convey information between processors starts to outweigh the advantage of adding more. For Keap1 (see Fig. 2 ▸), a molecular target for anti-inflammatory and antioxidant drug design (Cuadrado *et al.*, 2019 ▸), it is possible to obtain ∼330 ns per day using the GPU version of *AMBER*18 on a standard RTX2060 Nvidia graphics card (MD simulations of Keap1 contain around 30 500 atoms when solvated). For comparison, di­hydrofolate reductase (which contains around 23 000 atoms when solvated) runs at a speed of 85 µs per day on the specialized Anton 2 supercomputer architecture (Shaw *et al.*, 2014 ▸). In 2020 the UK HECBioSim consortium performed a comprehensive benchmarking exercise for popular MD codes (for example *NAMD*, *AMBER* and *GROMACS*), including HPC architectures that have not yet been used extensively for MD, such as ARM (see https://www.hecbiosim.ac.uk/access-hpc/our-benchmark-results/dirac-arm-benchmarks and https://www.hecbiosim.ac.uk/access-hpc/our-benchmark-results/isambard-benchmarks) and IBM Power 9 (see https://www.hecbiosim.ac.uk/access-hpc/our-benchmark-results/bede-benchmarks). This exercise highlighted that GPU versions of *GROMACS* and *AMBER* performed particularly well (see https://www.hecbiosim.ac.uk/access-hpc/our-benchmark-results/jade2-benchmarks), showing how the optimization of codes for new computational architectures such as GPUs can be transformative in terms of computational speeds.

Biomolecular simulations are stochastic because atomic motion is driven by random thermal noise. Minor perturbations to the starting conditions, such as swapping around the atomic speeds at the beginning of the simulation, will result in subtly different simulation trajectories, and structures, being sampled from the same phase space. To account for this inherent randomness, practioners run ‘repeat’ calculations from arbitrarily different starting conditions to generate a statistical ensemble. Comparing simulation trajectories, for example two simulations run with different force fields, is therefore challenging, because converged statistical averaging is needed to detect any discrepancies. However, replica calculations have the advantage that each runs concurrently, so achieving tenfold better sampling does not require waiting ten times longer for the simulation to finish, assuming that sufficient computational resources are available.

Pharmacological activity is sensitive to drug-binding kinetics as well as thermodynamics. Kinetics is relevant to factors such as clinical indication of the therapy and the duration of the therapeutic effect. The distinctive roles of thermodynamics and kinetics in drug discovery have been explained in a recent review (Tonge, 2018 ▸). In principle, on and off rates will be calculable using atomistic MD simulations when simulations are fast enough to observe multiple binding–unbinding events, simply by observing binding/unbinding kinetics within the MD trajectories. However, in practice advanced sampling methods need to be employed (Bruce *et al.*, 2018 ▸). The binding kinetics of representative biomolecular inter­actions, for example the biotin–strept­avidin interaction, the saquinavir–HIV1 protease interaction and the DOT1L–aminonucleoside inhibitor interaction, show that the on rates for ligand binding are relatively consistent (in the range 10^6^–10^8^ m^−1^ s^−1^) but the off rate varies with the dissociation constant of the interaction (*k*_off_, 10^−6^–10^2^ s^−1^; *K*_d_, 10^−14^–10^−6^ *M*). This implies that to observe unbinding events simulations of 0.01–100 000 s in length are required (Copeland, 2016 ▸), which are currently computationally unfeasible. Chemically activated conformational changes, for example in membrane-bound transporters, occur over millisecond timescales, and molecular motor timescales, which often additionally involve negotiating a complex, crowded cellular environment, can take minutes. The possible solutions are to either speed up the calculations or simplify the problem (see Table 2 ▸).

For the foreseeable future, multi-scale methods and enhanced sampling will be required, especially for larger systems such as protein–protein interactions. A robust comparison of current state-of-the-art methods for calculating protein–ligand association and dis­association rates against two well characterized benchmark systems (mutant T4 lysosyme–ligand and N-HSP90–inhibitor complexes) showed that simulations can already usefully predict relative dissociation rates, but emphasized that access to high-quality experimental data sets is essential for further methods development and validation (Nunes-Alves *et al.*, 2020 ▸). Advanced sampling applied to G-protein coupled receptors has enabled the complex conformational landscape to be reconstructed, providing structures of previously unseen active intermediates and revealing state-dependent cholesterol hotspots that are potential allosteric regulatory sites (Lovera *et al.*, 2019 ▸).

### 2.2. MD force-field parameterization is crucial for accuracy   

The accuracy of an MD simulation depends critically on the accuracy of the underlying energy model (force field; Dauber-Osguthorpe & Hagler, 2019 ▸), because this is how the relative energies of each molecular conformation are calculated. Force-field development is highly challenging because the potential must be carefully refined by balancing numerous parameters for every chemical motif of interest. As yet, no systematic automated method has emerged which performs this task satisfactorily, placing severe limitations on the reliability of computational predictions for pharmaceutical molecular design, especially for molecular-recognition events. The equilibria that govern binding and unbinding events in molecular recognition are exquisitely sensitive to small changes in the underlying free energy (Foloppe & Hubbard, 2006 ▸), since there is an exponential relation between the association binding constant and the corresponding binding free energy (Fig. 1 ▸). A 1 kcal mol^−1^ free-energy change results in an almost tenfold change in the corresponding binding constant (Foloppe & Hubbard, 2006 ▸). Consequently, very small errors in the calculated binding potential energies (and the accompanying free energies) result in exponentially magnified errors in the binding constants, *i.e.* unreliable predictions of ligand–target affinities. Thus, somewhat quantitative binding-affinity predictions would require force fields that are accurate to at least 0.5 kcal mol^−1^. Unfortunately, the complexity and diversity of intermolecular interactions has made such accuracy elusive. This issue has plagued binding-affinity calculations (Mikulskis *et al.*, 2014 ▸), especially when confronted with the vast diversity of small molecules investigated for drug discovery. There is no fundamental obstacle to the derivation of a force field covering the vast array of chemistries encountered in pharmaceutical discovery, apart from the tremendous determination and effort required. This is being tackled by some research groups, with incremental but steady progress (Vanommeslaeghe & MacKerell, 2015 ▸; Harder *et al.*, 2016 ▸; Hagler, 2019 ▸; Piana *et al.*, 2020 ▸). Alongside improved configurational sampling, this provides a stronger foundation for molecular simulations to contribute to pharmaceutical research.

### 2.3. Current applications of biosimulation in pharma   

Many small molecules are difficult, time-consuming or resource-intensive to make synthetically. Simulations capable of predicting biomolecular binding free energies reduce the number of compounds that need to be synthesized and tested in the laboratory, improving the efficiency of the drug-discovery pipeline. Free-energy perturbation (FEP) has begun to be used by pharma to predict the relative binding free energies of congeneric compounds (Jorgensen, 2009 ▸; Wang *et al.*, 2015 ▸; Schindler *et al.*, 2020 ▸). Most commonly, FEP calculations morph one ligand (or interacting residue in the binding site) into another using a series of small alchemical changes (Michel *et al.*, 2010 ▸). This can be computationally expensive because the perturbation must be applied slowly to obtain adequate sampling. FEP is successful as a theory since it is based on a sound statistical-mechanical treatment, can be implemented computationally and is adapted to the medicinal chemistry practice of introducing stepwise modifications to lead compounds during optimization. FEP considers local perturbations resulting from small chemical changes to the ligand or its binding pocket. This reduces the computational complexity of the calculations, because it does not need to either predict the relative affinity of chemically diverse ligands or identify *de novo* binding modes/sites, or sample large-scale conformational rearrangements of the protein. Moreover, by focusing on a single chemical scaffold, researchers can tune their parameterization to achieve the accuracy necessary, without the need to provide a general solution for the whole of chemical space. In addition, FEP can be used to select mutations to engineer protein stability (Duan *et al.*, 2020 ▸; Ford & Babaoglu, 2017 ▸); such stabilized proteins are sought for more robust assays, increased chances of crystallization or more stable therapeutic biologics.

Simulations in drug discovery go well beyond FEP calculations. MD simulations have been used to identify binding hotspots on protein surfaces via so-called co-solvent simulations (Ghanakota & Carlson, 2016 ▸), in which a protein is simulated in the presence of selected small solutes present at high concentrations in aqueous solution; it can identify protein surface patches with a propensity to bind organic fragments (in competition with water), or highlight the type of chemical group displacing water efficiently in a particular pocket. Since the surface of a protein is dynamic, some pockets open only transiently and may not be observed in an X-ray structure, and for this reason have been dubbed ‘cryptic pockets’ (Vajda *et al.*, 2018 ▸). Even a small cryptic pocket may be of interest if it can be reached from a nearby larger binding site, in particular when targeting shallow protein sites involved in protein–protein interactions. Cryptic pockets may be revealed by standard MD (Martinez-Rosell *et al.*, 2020 ▸) or enhanced sampling methods (Oleinikovas *et al.*, 2016 ▸). The same approaches may also reveal allosteric pockets, and allosteric modulation of proteins (through long-range changes in structure or dynamics; Motlagh *et al.*, 2014 ▸), which have been a focus of the pharmaceutical industry in recent years (Durrant & McCammon, 2011 ▸). MD simulations in explicit solvent can also be used to observe the conformational flexibility of compounds in their unbound state (Foloppe & Chen, 2016 ▸) to approach the energetic and entropic contributions of compound conformational focusing upon binding to a biomolecule. The prediction of compound permeation across lipid membranes, which is a physicochemical property vital to both drug uptake, distribution and toxicity, is yet another promising application of simulations (Awoonor-Williams & Rowley, 2016 ▸).

The ability to examine the dynamic structure of a protein via plain MD should not be underestimated. Visualizing side-chain rearrangements in a targeted site or the dynamics of nearby loop conformations can provide mechanistic insight that is invaluable for drug development, for example in the analysis of antivirals against influenza (Amaro *et al.*, 2009 ▸). Indeed, simulations have been likened to a ‘computational microscope’ (Dror *et al.*, 2012 ▸). While simulations have begun to contribute to molecular design in the pharmaceutical and biotechnology industries, other engineering fields, such as aerospace, have benefitted more rapidly from the adoption of computational tools. The developments needed to bring more computational tools into pharma are summarized in Table 3 ▸.

## 3. The future potential of biomolecular simulation for pharma   

All molecular recognition in biology fundamentally involves chemical complementarity, molecular flexibility and the surrounding solvent environment. Physics-based simulations, such as MD, are uniquely able to capture the details of this physical chemistry because these models are built up from a physical understanding of molecular interactions and mechanics. They have the capability to capture both atomistic details and global conformational changes. Other (very useful) computational chemistry approaches include docking using empirically derived scoring functions, quantitative structure–activity relationship analyses and quantum-mechanical calculations. However, these approaches are unable to represent the full complexity of binding phenomena in aqueous solvent, because when used in isolation these methods are unable to account for dynamics or hydration of the compounds and the protein. Therefore, it is essential that drug-discovery teams learn how to harness the growing power of physics-based simulations. In addition to drug-design applications, this should provide much-needed theoretical insights into molecular recognition. An essential additional development must be the improvement of the potentials (‘force fields’) used to propagate the simulations. As argued above, much of the groundwork for this is being laid, and progress towards those objectives is achievable in the foreseeable future. However, cultural shifts will also be required alongside technical improvements.

The time taken to obtain results is an important criterion because of the fast pace of industrial drug-discovery projects. The rapid response of the biomolecular simulation community to the COVID-19 pandemic shows that MD is now fast enough to provide insightful results as the situation evolves. MD simulations combined with cryo-EM have identified a linoleic acid binding site in the SARS-CoV-2 spike glycoprotein, which offers a new target site for drug design (Toelzer *et al.*, 2020 ▸); simulations of emerging mutations in the receptor-binding site of the spike protein have provided molecular-level insights into the associated changes in transmissibility (Luan *et al.*, 2021 ▸) and MD studies of spike-protein glycoforms have shown how much of the surface is shielded by glycans, with implications for antibody recognition and design (Grant *et al.*, 2020 ▸).

As biomedical interventions become more sophisticated, for example using antibody–drug conjugates, smart drug-delivery vehicles, theranostics or other biologics, fundamentally new types of computational models to optimize design will be needed. Computer models constructed to complement experimental studies help researchers to visualize the different components of their experimental procedures, which can assist in identifying variables that need to be controlled. The success of the *AlphaFold* neural network in predicting protein structures (Senior *et al.*, 2020 ▸) has generated much interest in applying artificial intelligence to pharmaceutical design (Schneider *et al.*, 2020 ▸); however, past experience also suggests that overenthusiasm for nascent tools can lead to disappointment (Jordan, 2018 ▸). Engineering capabilities have been enhanced by computer models throughout industry, and in Part 2 we will discuss how developments in computer hardware, software and methods for standardization and validation have enabled aerodynamics and weather modelling to become embedded in the research culture in these fields. Quantitative biomolecular simulations promise equivalent benefits and may not be far behind.

## Supplementary Material

Supplementary Movie for Figure 1. DOI: 10.1107/S2059798321009712/qr5005sup1.mp4

## Figures and Tables

**Table 1:** Caveats for PDB structural information; the PDB (and increasingly the EMDB) are essential resources for structural molecular biology

*Protonation states*. As most X-ray structures do not resolve hydrogen, the protonation states of titratable amino-acid residues are generally unknown. NMR can allow specific protonation states to be assigned, although this is complex, low-throughput and system-dependent. Metal ions are also difficult to identify with certainty [X-ray absorption fine structure (XAFS) or anomalous data collection at specific wavelengths are required]. This has implications for drug design because the nature of the ion (for example zinc, manganese or magnesium) matters if the compound binds to it directly.
*Flexible loops*. X-ray structures frequently do not resolve the most flexible regions of biomolecules, such as unstructured protein loops. Side chains or loops with multiple fixed conformations can also be invisible unless very high-resolution diffraction data are collected. Highly flexible structures, such as intrinsically disordered proteins or single-stranded nucleic acids, will not crystallize at all.
*Post-translational modifications*. Many proteins are chemically modified during the course of their function, for example through the addition of sugars (glycosylation), lipid modifications that target proteins to the membrane (lipidation; for example farnesylation), phosphorylation and many others. These modifications can be permanent, such as glycosylation of the collagen protein, or transient, such as the tagging of proteins for destruction by ubiquitination. This chemical diversity is often vital to ensure that proteins follow the correct biochemical pathways or perform their structural roles, but is difficult to capture through structural determination.
*Environmental conditions*. Crystallization conditions are nonphysiological, and sometimes exceedingly so, with non-natural mutations, extremes of pH, salt concentration and nonbiological organic additives and even cross-linkers. For membrane proteins, this is particularly problematic because the number of factorial conditions screened for initial crystal hits (often 1000 different conditions for soluble proteins) are multiplied by the variety of detergents and/or lipids that need to be screened. Protein–protein contacts in the crystal can also potentially distort the shape of a protein or that of a bound compound, or more likely, lock the protein into a single conformation from its dynamic conformations in solution. *In vivo*, proteins are often organized into functional multi-unit complexes (Robinson *et al.*, 2007 ▸), which may influence their conformations, and which also bury key surfaces.

**Table 2:** Opportunities for improving the predictive power of biomolecular simulations Improvements to both speed and accuracy are ongoing, and are interdependent. Faster calculations and better sampling improve the statistical convergence of the simulations, which makes the assessment of the underlying energy models more reliable.

*Hardware and algorithm speed*. Improvements to the mathematical structure of the algorithms used to propagate the dynamics, such as optimization on bespoke hardware, can massively accelerate progress, as has been demonstrated by Shaw *et al.* (2014 ▸). Continuous improvement in computing hardware has brought the sampling capability of MD simulations and variants into a regime which is now relevant to a range of molecular-design questions in drug discovery (De Vivo *et al.*, 2016 ▸). Improved MPI parallelization, and porting of simulation codes such as *AMBER* and *GROMACS* to GPUs, have already brought massive improvements in speed to the user community. Changes to algorithms that enable better exploitation of larger (for example, towards exascale) parallel resources could also be hugely beneficial in the future.
*Improving sampling efficiency*. The simulation community has created a wealth of tools to accelerate conformational sampling (for an accessible review, see Lazim *et al.*, 2020 ▸). Replica exchange MD, for example, uses elevated temperatures to push biomolecules over the conformational barriers that trap simulations in local minima. Techniques such as metadynamics use biasing potentials to prohibit molecules exploring conformations that have already been adequately sampled. Alternatively, adaptive MD exploits successive MD trajectories where their starting configurations depend on the previous exploration of conformational space (Lovera *et al.*, 2019 ▸).
*Simplified models*. Major efforts are directed to provide less computationally intensive models, such as coarse-grained simulations, Gaussian/elastic network models and Brownian dynamics simulations. Coarse-grained simulations require fewer particles (Kmiecik *et al.*, 2016 ▸), can use a longer integration time step and have proven to be particularly successful for studying membrane proteins, including their interactions with ligands (Souza *et al.*, 2020 ▸; for a review of MD simulations of lipid–protein interactions, see Muller *et al.*, 2019 ▸). Gaussian/elastic network models have shown the relationship between structure and dynamics, and how this can be used to understand how functional protein conformational changes occur (Tobi & Bahar, 2005 ▸), including allosteric interactions (McLeish *et al.*, 2015 ▸). Brownian dynamics (Huber & McCammon, 2019 ▸) has been used in conjunction with atomistic methods to predict binding and unbinding kinetics (Jagger *et al.*, 2018 ▸) and to investigate molecular crowding (McGuffee & Elcock, 2010 ▸). To achieve this improved efficiency, all of these simplified methods need to impose conformational restrictions on the flexibility of the protein. Such simplifications involve tradeoffs with respect to atomistic accuracy. Improved theoretical understanding of the mechanics of proteins and their large-scale conformational changes, and of the role of hydration, could potentially lead to fundamentally new models capable of exploring a wider diversity of protein conformations relevant to function, and may be essential to further our understanding of large macromolecular complexes.
*Improving accuracy*. Atomistic and coarse-grained simulations rely on empirically derived parameters chosen to capture essential chemical details in protein interactions, without the need to explicitly represent electronic structure. Force-field improvement requires continued efforts and access to high-quality experimental information for validation. In pharma, a big issue is the development of accurate force fields for diverse chemical compounds. The capability to perform simulations of small proteins for multiple microseconds now provides statistically converged trajectories, which will allow force-field deficiencies to be distinguished from sampling limitations; for example the folding free energy of Trp cage mutants (Piana *et al.*, 2020 ▸). Despite continued improvements, it is likely that general-purpose atomistic force fields will be inadequate for describing certain classes of biomolecular interactions, such as those involving unusually high levels of electronic polarization. Polarizable force fields go beyond conventional fixed-charged models in treatment of local electrostatic interactions, and for example have been shown to improve descriptions of ion permeation through membrane-bound protein ion channels (Jing *et al.*, 2019 ▸). Hybrid quantum-mechanical/molecular-mechanical (QM/MM) calculations, in which a specific region (for example a substrate-binding site) represented at the QM level is embedded within a larger classical system, have also been used to study polarization effects (Beierlein *et al.*, 2011 ▸), including a systematic study of the polarization of ligands by protein targets (Willow *et al.*, 2020 ▸). As conventional classical force fields are not able to represent rearrangements of covalent bonds, studying enzyme-catalysed reactions requires either quantum mechanics or specialist methods [for example multi-configurational reactive MD (Yamashita *et al.*, 2012 ▸) or the empirical valence-bond model (EVB; Kamerlin & Warshel, 2011 ▸)].

**Table 3:** Developments that may encourage the adoption of biomolecular simulations by industry

*Standards validation and software reliability*. Industry has more confidence in computational tools when there is standardization, consensus on best practice and error quantification. For experimental drug development, for the foreseeable future, computational tools provide a route to speeding up the initial discovery stage rather than truncating the process and providing a direct route from *in silico* design to clinical evaluation. Therefore, the current priorities relate to barriers to entry, robustness and effectiveness of the software for a diverse user group. Currently, there is not a clear standout docking and simulation pipeline that is able to achieve all of these objectives across the full range of molecular targets. Whilst improving docking algorithms and scoring functions has many challenges, rapid progress in the tests of MD force fields could be possible now that statistical convergence is sufficient for comparison with experimental data. However, this requires the experimental and simulation communities to work together to generate the experimental data sets that are needed to validate the models. The Drug Design Data Resource Grand Challenge aims to ‘test and advance the state of the art in protein-ligand modelling by holding community-wide, blinded, prediction challenges’ (Gaieb *et al.*, 2019 ▸). These competitions inspire communities to define objective criteria for assessing the performance of computational predictions, and reveal the most promising approaches. This has the potential to assist industry in making informed choices about the computational tools that will provide the greatest benefits to their research programs. Community-supported, standardized software repositories and data sets for validating simulations could therefore be highly beneficial, because new methods could be benchmarked for reliability, ease of use, speed and accuracy at the time of publication.
*Ease of use of software*. In academia, a successful methodology often emerges as a result of numerous incremental improvements and adjustments contributed by multiple research teams. This results in a myriad of complementary tools that may perform much the same function, but using slightly different assumptions or parameters. For industry, the effort required to assess performance is then prohibitive. Moreover, poor reliability of software installations, which can involve complex inter-dependencies on external tools, licencing obstacles, limited documentation and sustainability issues (for example outdated software libraries) also discourages industry uptake of new computational tools from academic groups.
*Error quantification*. Reduced computational costs and development of automated workflows has now enabled simulators to explore how errors in stochastic trajectories can be quantified (Grossfield *et al.*, 2018 ▸). In one approach aimed at drug design, multiple instances of a simulation with different starting conditions were performed, and the error was calculated from the ensemble distribution (Bhati *et al.*, 2018 ▸). The propagation of errors across different computational regimes is also important in multi-scale simulations, and will need careful assessment when such schemes are used to model biomolecular complexes.
*Multi-scale and integrative modelling*. The revolution in molecular biology generated by electron cryo-microscopy and electon cryo-tomography is generating structural information for ever-larger biomolecular complexes. These experiments are revealing that biomolecular structure is highly organized across all length scales and why the cellular context of biomolecules is important to their function and consequently for their pharmacological responses (Robinson *et al.*, 2007 ▸). Minimal coarse-grained models have been used to simulate super-macromolecular structures such as the cytoskeleton, whole genomes and protein compartments (Hafner *et al.*, 2019 ▸). They are increasingly used for integrative biology, in which simulations incorporating experimental restraints are performed at multiple resolutions, so that disparate sources of experimental information can be combined into a consensus model (Koukos & Bonvin, 2020 ▸). One challenge for biomolecular simulation is our lack of understanding of the multiple variables in biological systems, and the implications for the accuracy required for molecular-level calculations. Coarse-grained simulations that explore the next length scale up will provide better understanding of the sensitivity of biological mechanisms to specific atomistic details, providing guidance on accuracy requirements.
*Cultural issues*. It takes time for novel methods to be accepted, especially if that requires a shift in mindset. Pre-clinical discovery proceeds primarily by wet-laboratory trial and error, with high attrition rates being the norm. Theoretical predictions can frequently appear futile when confronted with the complexities of chemistry and biology. Thus, the notion that MD simulations may offer respectable, or even operational, science still faces prejudice ranging from scepticism to hostility (Merz, 2010 ▸; Mikulskis *et al.*, 2014 ▸; Lowe, 2019 ▸). Well documented credible exemplars are essential to educate and convince. Such studies have started to surface, and several pharma companies are investing in a computational infrastructure intended for physics-based molecular simulations. Raising awareness about the scientific foundations of molecular simulations, and the inclusion of computational chemists in decision making, may encourage the wider integration of computational modelling with experimental planning by pharma.
